# A new type of calcium-rich biochars derived from spent mushroom substrates and their efficient adsorption properties for cationic dyes

**DOI:** 10.3389/fbioe.2022.1007630

**Published:** 2022-09-20

**Authors:** Haibo Zhang, Long Su, Caiping Cheng, Hongyan Cheng, Mingchang Chang, Fenwu Liu, Na Liu, Kokyo Oh

**Affiliations:** ^1^ College of Resources and Environment, Shanxi Agricultural University, Taigu, China; ^2^ State Key Laboratory of Sustainable Dryland Agriculture (in preparation), Shanxi Agricultural University, Shanxi, Taiyuan, China; ^3^ Shanxi Key Laboratory of Edible Fungi for Loess Plateau, Taigu, China; ^4^ College of Basic Science, Shanxi Agricultural University, Taigu, China; ^5^ College of Food Science and Engineering, Shanxi Agricultural University, Taigu, China; ^6^ Collaborative Innovation Center of Advancing Quality and Efficiency of Loess Plateau Edible Fungi, Taigu, China; ^7^ Center for Environmental Science in Saitama, Kazo, Japan

**Keywords:** adsorption mechanism, biochar, calcium, cationic dye, spent mushroom substrate

## Abstract

Adsorption is commonly accepted as a most promising strategy in dye wastewater treatment, and the widespread use of adsorption emphasizes the need to explore low-cost but excellent adsorbents. Herein, a low-cost adsorbent (calcium-rich biochar) was developed, which was directly pyrolyzed from spent mushroom substate without any modification. This study evaluated the potential application of two calcium-rich biochars (GSBC and LSBC) derived from spent substrates of *Ganoderma lucidum* and *Lentinus edodes*, respectively. The effects of pyrolysis temperature on the calcium-rich biochars characteristics and their adsorption mechanism for cationic dyes (Malachite Green oxalate (MG) and Safranine T (ST)) were studied systematically. The increase in pyrolysis temperature from 350 to 750 °C led to an increase in both biochar ash, Ca content, and specific surface area, which made high-temperature biochars (GS750 and LS750) the superior adsorbents for cationic dyes. Batch adsorption results showed LS750 was more efficient to adsorb dyes than GS750 attributed to its higher Ca content and larger specific surface area. According to the Langmuir model, LS750 had high adsorption capacities of 9,388.04 and 3,871.48 mg g^−1^ for Malachite green and ST, respectively. The adsorption mechanism of dye MG could be attributed to pore filling, hydrogen bonding, electrostatic interaction, ion exchange, and π-π stacking, while ST adsorption mainly involved pore filling, electrostatic interaction, ion exchange, and π-π stacking. Attributed to their excellent adsorption performance, cheap source, and good reusability, biochars obtained from SMSs were very promising in dyeing wastewater treatment.

## 1 Introduction

With the rapid development of paper, food, and textile industries, dye wastewater was inevitably discharged into the natural water (Mustafa and Hayder 2021). It is estimated that more than 7,000,000 tons of commercial dyes is produced annually (Azari et al., 2020). These pollutions pose seriously threat to the natural environment and public health. Among them, Malachite green (MG) and Safranine T (ST) are most vastly used azoic cationic dyes (basic dyes) (Gurbani et al., 2012; Tsai et al., 2022). MG (basic green), prepared from benzaldehyde and dimethylaniline, is commonly used as a direct dye for silk, wool, jute, paper, pharmaceutical and leather products (Arora et al., 2020). It is also applied to treat protozoal and infections of fish and fish eggs as well as *Oomycete* (*Saprolegnia*) infecting commercial aquaculture (Wu et al., 2022). For these purposes, thousands of tons of MG and associated triarylmethane dyes are produced each year thereby causing potential health risk to humans (Arora et al., 2020). ST (basic red) has been used in many textile products such as cotton, Liberian fibers, homespun, silk, lashing and sheet (Ugraskan et al., 2022). This pollutant causes severe symptoms in humans like loathing, puke, hypertension and diarrhea (Ugraskan et al., 2022). So eliminating MG and ST dyes from wastewater is inevitable and necessary.

Up to now, many researchers have endeavored to remove dyes from wastewater (Ravindiran et al., 2019). The main methods utilized for dye wastewater treatment are chemical coagulation, membrane separation, solvent extraction, photocatalytic oxidation, electrolysis, microbial degradation and adsorption (Sajjadi et al., 2021; Yar and Parlayici, 2022; Zhao et al., 2022). Among them, adsorption is low-cost, easy to operate and highly efficient, and the adsorption process neither changes the structure of the dye nor produces secondary pollution, thus it is commonly accepted as a promising strategy for the removal of dyes in wastewater (Zhu et al., 2020). Many adsorbents have been exploited, including minerals (Anastopoulos et al., 2018), carbon materials (Sutar et al., 2022), and biomass (Sartape et al., 2017). However, the widespread use of adsorption emphasizes the need to explore low-cost and high-efficiency adsorbent.

Owing to low cost, simple processing, and eco-friendly, biochar obtained by incomplete pyrolysis of carbon-rich materials has attracted increasing attention in dyeing wastewater treatment (Han et al., 2019). However, pristine biochars from common-used agricultural wastes usually have limited adsorption capacities for dyes (Patra et al., 2021). To improve adsorption capacity, activation and functionalization techniques (e.g., steam, acid, alkaline, metal salt, oxidant, organic solvents, and nitrogenous compounds) were used to alter biochar physicochemical properties (Hokkanen et al., 2016; Medeiros et al., 2021; Tsai et al., 2022). But for costly and tedious operation, most techniques were limited to use in research laboratories and rarely applied on a large scale. Thus, biochars pyrolyzed directly from pure agricultural wastes with superior adsorption capacity need to be further explored.

Calcium-rich material is recently highlighted as a promising adsorbent for dyes (Dai et al., 2018). For instance, waste eggshell, which contains large amounts of calcium salts, demonstrated the efficacy in adsorbing methylene blue and Congo red in wastewater (Abdel-Khalek et al., 2017). The CaO-doped ZrO_2_ nanoparticles were regarded as the brilliant adsorbents for the toxic azo dye of Congo red (Singh et al., 2022). The crab shell-derived biochar (mainly including calcite) showed super adsorption capacities of Malachite green and Congo red, respectively (Dai et al., 2018). The role of these calcium minerals is to enable a chemical interaction via ion exchange and/or electrostatic adsorption between the targeted dye and adsorbent (Dutta et al., 2021). Thus, both calcium minerals and carbon fractions in feedstock may synergistically determine the physicochemical properties of biochars, and eventually influence their adsorption performances (Buss et al., 2016). A typical feedstock, spent mushroom substrates (SMSs) are from residual culture substrates of the edible fungus industry. China is the world’s largest producer and exporter of edible mushrooms. Large amounts of SMSs (approximately 5 kg) are generated during the production per kg of edible fungi (Chang et al., 2020). According to statistics, the total yield of SMSs in China reached approximately 150 million tons each year (Gao et al., 2021). Therefore, utilizing SMS resources scientifically and reasonably have become a critical issue. SMSs contains a wide variety of organic materials, including woody biomass, crop straw, animal wastes, cotton seed hull, wheat bran, corncobs, etc. (Sahithya et al., 2022). In preparation, calcium-containing minerals (e.g., gypsum and lime power) are commonly added to the culture substrate to provide mineral nutrition and neutralize acidic materials generated from fungal metabolites and sterilization (Chang et al., 2020). After harvest, calcium constituents were enriched in SMSs which attributed to both biomass-inherent and exogenous additives in the culture substrate. Therefore, SMSs might be served as an ideal raw material for the preparation of calcium-rich biochars, and their physicochemical characteristics and adsorption performance were worthy of further investigation. However, few studies can be found that have comprehensively investigated the adsorption of MG and ST dyes by SMS derived biochars.

In this work, two types of SMSs (GSBC and LSBC) from widely cultivated mushroom species in China (*Ganoderma lucidum* and *Lentinus edodes*) were collected as biochar feedstock for the dye removal. Two typical cationic dyes of MG and ST were chosen as the model pollutant. The objectives of this research are 1) to compare the characteristics of two SMSs-biochars at pyrolysis temperatures of 350, 550, and 750°C, 2) to systematically analyze the adsorption performance through pH effects, adsorption kinetics and isotherms, 3) to reveal the adsorption mechanism of SMSs-biochars for cationic dyes in depth, and 4) to study their desorption capacities.

## 2 Materials and methods

### 2.1 Materials

Two types of SMSs including *Ganoderma lucidum* spent substrate (GS) and *Lentinus edodes* spent substrate (LS) were obtained from Edible Fungi Center, Shanxi Agricultural University, China. They were washed with deionized water several times, dried at 60 °C overnight, crushed with a grinder (SL-100, Zhejiang, China), and finally sieved through a sieve with 0.5 mm meshes. The obtained samples were stored in vacuum-sealed bags for future use. All chemicals like Malachite Green oxalate (MG) and Safranine T (ST) were analytical grade and purchased from Hengxing Chemical Reagent Manufacturing Company, Tianjin.

### 2.2 Preparation of biochar

The biochar was prepared by slow pyrolysis with limited oxygen in a tube furnace (STG-60-12, Chongqing Songlang Inc., China). SMSs were pyrolyzed in a N_2_ atmosphere at the target temperature of 350, 550, and 750°C for 3 h with the heating rate of 15°C min^−1^ before cooling to room temperature. Then the obtained material was ground through a sieve with 100 meshes (0.15 mm). The different biochars are abbreviated by source as GSBC (spent *Ganoderma lucidum* substrate) and LSBC (spent *Lentinus edodes* spent substrate).

### 2.3 Biochar characterization

The yield and pH of biochars were measured according to the method described by Chang et al. (2020). The ash content was determined by a muffle furnace heating at 800°C for 1 h (Kwak et al., 2019). The content of C, H, and N in the samples was determined using an element analyzer (Elementar Vario MACRO Cube, Germany). The surface morphology of biochars was observed using a scanning electron microscopy (SEM) instrument (FEI Inspect F50, USA). The specific surface area (*S*_BET_) was obtained based on Brunner-Emmet-Teller (BET) theories. The pore size was estimated based on Barrett–Joyner–Halenda (BJH) method by specific surface and pore size analyzer (TriStar II 3020, United States) with nitrogen adsorption at 77 K. X-ray diffractometry (XRD) (D8 advance, Japan) was employed for analysis of the mineral constituents on biochar surfaces by scanning at 2θ ranged from 10 to 70°. Fourier transform infrared spectroscopy (FTIR) (Fourier 27, Germany) was used to identify surface functional groups in the 4,000–500 cm^−1^ range with a resolution of 4 cm^−1^.

### 2.4 Batch adsorption experiments

Batch adsorption experiments were conducted for the removal of MG and ST from an aqueous solution using GSBC and LSBC, respectively. Specifically, 15 mg of biochar was placed in a 50 ml centrifuge tube followed by addition 40 ml of designed solutions. And then, the mixture samples were stirred at 250 rpm for 1,440 min. Additionally, to investigate the pH effect on adsorption characteristics of dyes, the initial pH was adjusted by diluted NaOH and HCl to the range of 3–10 for MB (3,500 mg L^−1^) and ST (3,500 mg L^−1^). For the batch kinetic studies, samples were taken out and the remaining dye concentration was determined at various time intervals of 10–1,440 min at the optimum pH. For the determination of adsorption isotherms, the initial concentrations varied from 250–3,500 mg L^−1^ for MG and ST over a 24 h contact period at the optimum pH. All adsorption experiments were conducted in triplicate and the average values were reported. Finally, the mixture was centrifuged at 4,000 rpm for 10 min and then filtered with 0.45 µm PTFE membrane. The filtrate was analyzed with a UV-vis spectrophotometer (UV-2500, SHIMADZU, Japan) for calculation of the residual dye concentration at 617 nm (MG) (Dai et al., 2018) and 554 nm (ST) (Wang et al., 2016), respectively.

The dye removal capacities of the two biochars were calculated by the mass balance equation:

$$Q_{e}=\frac{\left(C_{0}-C_{e}\right)V}{m}$$ (1)

where *Q*_e_ (mg g^−1^) represents the dye adsorption capacity of the biochar at equilibrium; *C*_0_ (mg L^−1^) and *C*_e_ (mg L^−1^) are the initial and equilibrium concentration of dye; *V* (L) is the volume of adsorbate solution, and *m* (g) is the mass of the adsorbent.

### 2.5 Empirical adsorption models

The data from the adsorption kinetics experiments were fitted with the pseudo-first order, pseudo-second order, and intra-particle diffusion model. The data from the adsorption isotherm model were fitted with the Langmuir and Freundlich isotherm models. All mathematical presentations of these models are listed in the Supplementary Information.

### 2.6 Desorption and reusability experiments

Desorption experiments were carried out with 95% ethanol for two dye-loaded biochars. All samples after sorption were separated from the mixture via centrifugation, and dried for 24 h at 70 °C. Then, 40 ml of eluent was prepared and transferred into a 50 ml centrifuge tubes including 15 mg of the dye-loaded biochar. The remaining procedure was detailed in section 2.4 as mentioned above. The recyclability of GSBC and LSBC were evaluated by four adsorption-desorption cycles for each dye.

## 3 Results and discussions

### 3.1 Material characterization

#### 3.1.1 Basic physicochemical properties

As shown in Table 1, the yields of GSBC and LSBC declined as the pyrolysis temperature increased from 350 to 750°C, which was consistent with the findings of Yuan et al. (Yuan et al., 2011) that the declined yield was closely associated with the loss of volatile organic compounds and water during pyrolysis. Both GSBC and LSBC prepared at high pyrolysis temperature owned a higher pH value due to the loss of acidic functional groups (e.g., carboxylic acids and phenols) and the release of alkaline minerals (Tang et al., 2019). Increased pyrolysis temperature caused the increased C content and the decreased H and N content in biochars. This is mainly because pyrolysis led to the H, O, and N escape from the carbon chain, then form H_2_O or volatile nitrogen-containing substances, eventually the accumulation of C in biochars (Tang et al., 2019).

**TABLE 1:** The physicochemical properties of GSBC and LSBC.

Samples	Yield (%)	Ash (%)	pH	Elemental analysis (%)						
				C	H	N	K	Ca	Mg	Na
GS350	44.13	12.18	7.78	53.23	9.46	2.55	0.88	4.26	0.55	0.29
GS550	35.79	19.92	10.2	54.68	9.38	2.49	0.92	6.38	0.68	0.47
GS750	27.86	21.30	11.38	55.46	9.28	2.38	1.26	8.94	0.72	0.66
LS350	47.88	23.40	8.52	52.39	9.42	2.39	1.03	7.48	1.09	0.52
LS550	39.75	26.05	10.48	53.61	9.39	2.38	1.12	9.66	1.09	0.73
LS750	29.00	29.62	11.59	54.36	9.38	2.37	1.25	11.35	1.12	0.92

Note: GS/LS350-750: the biochars produced from spent *Ganoderma lucidum* (GS) and *Lentinus edodes* (LS) at 350–750°C.

In addition, high temperature increased the ash content which was due to the inorganic minerals in biomass were not easily lost during pyrolysis. The ash content was 12.18, 19.92, and 21.30% for GS350, GS550, and GS750 and 23.40, 26.05, and 29.62% for LS350, LS550, LS750, respectively, indicating that there was higher inorganic mineral in LSBC than GSBC. Similarly, the content of K, Na, Ca, and Mg in biochars increased with the increased pyrolysis temperature. Among all mineral elements, Ca was the most abundant element accounting for more than 70% for all biochars. Apart from biomass-inherent minerals, Ca-rich features of these biochars were probably associated with exogenous additives in SMSs. For example, in mushroom cultivation, Ca-containing minerals (e.g., gypsum and lime power), are usually added to the culture medium to provide mineral nutrition and/or neutralize acidic materials which are caused by the fungal metabolites and the sterilization process (Chang et al., 2020). Compared with GS750, LS750 had higher Ca content, which was consistent with the changes of its ash content and pH value, implying there was more Ca-containing minerals input during *Lentinus edodes* cultivation.

#### 3.1.2 BET analysis

**FIGURE 1:**
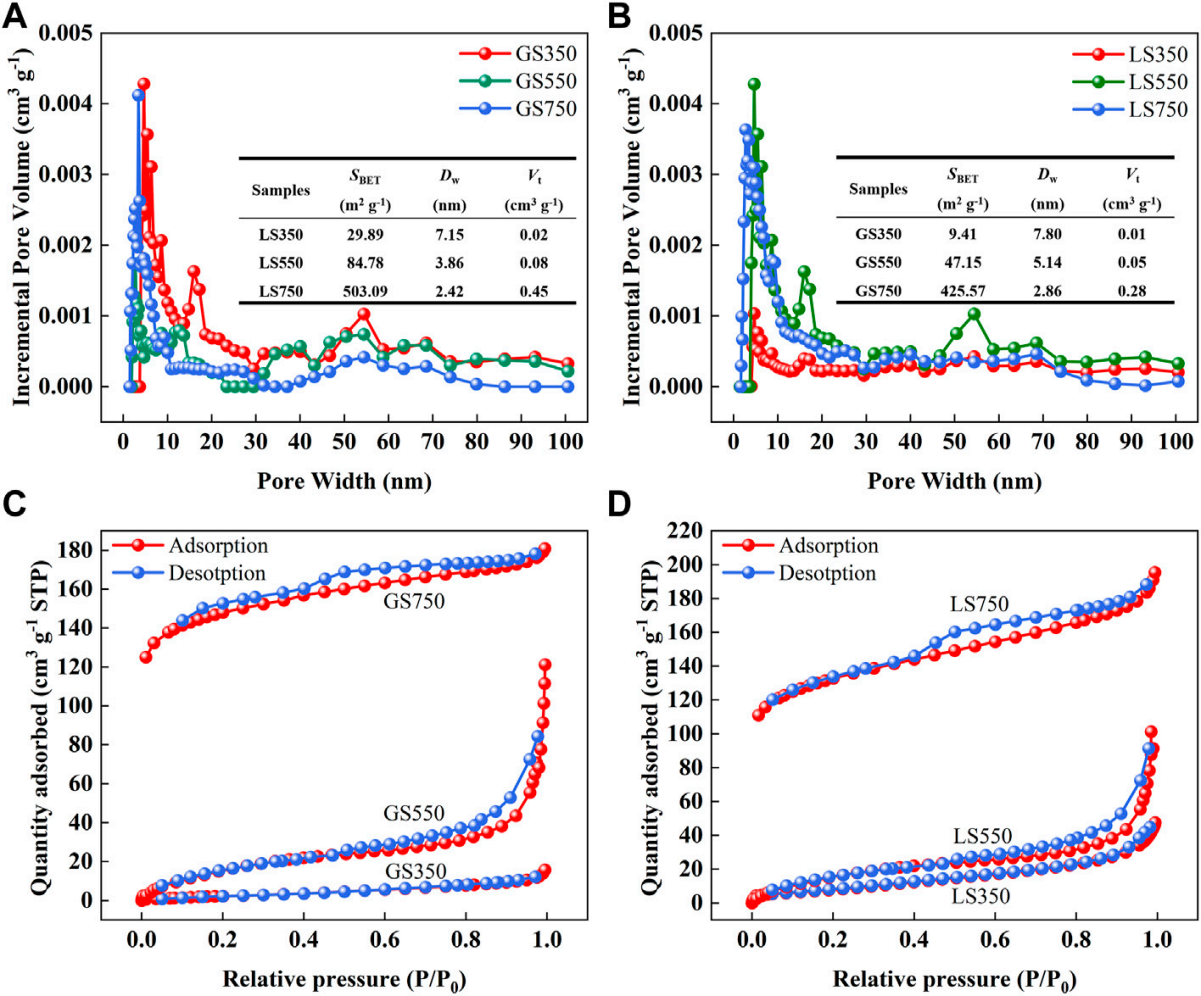
The pore-size distribution curves of GSBC **(A)** and LSBC **(B)**, and N_2_ adsorption-desorption isotherm of GSBC **(C)** and LSBC **(D)**. Insets in **(A)** and **(B)** are the data of specific surface area (*S*_BET_), average pore diameter (*D*_w_), and total pore volume (*V*_t_) of biochars.

The pore-size distribution curves, specific surface area (*S*_BET_), total pore volume (*V*_t_), and average pore diameter (*D*_w_) of GSBC and LSBC were shown in Figures 1A,B. Pyrolysis greatly enhanced *S*_BET_ and *V*_t_ of GSBC and LSBC due to the removal of volatile components (e.g., benzene derivatives, ethylene, and hydrogen) from the carbon skeleton (Sun et al., 2014). Conversely, the elevated pyrolysis reduced the *D*_w_ of both biochars thus enlarged their *S*_BET_. SL750 had the larger *S*_BET_ (503.09 m^2^ g^−1^) than that of GS750 (425.57 m^2^ g^−1^). The larger *S*_BET_ can provide more sufficient adsorption sites for dyes, thus is commonly considered to be more conducive to sorption (Ahmad et al., 2012).

Figures 1C,D showed the N_2_ adsorption-desorption isotherms of GSBC and LSBC. The isotherm of both GS350 and LS350 conformed to the type II isotherm defined by IUPAC, indicating their non-porous properties, which was consistent with the low *S*_BET_ of GS350 (9.41 m^2^ g^−1^) and LS350 (29.89 m^2^ g^−1^). This is probably due to the incomplete pyrolysis at low temperature hinders the formation of pores (Choudhary et al., 2020). Both biochars prepared at high temperature (≥550°C) belonged to type-IV N_2_ adsorption-desorption isotherm with the H_3_ (550°C) and H_4_ (750°C) type hysteresis loop, respectively, indicative of mesoporous structure properties (Hu and Hsieh 2017). This is in accordance with their *D*_w_ (2–50 nm) as shown in the inset of Figures 1A,B. Therefore, high-temperature biochars show well-developed mesoporous structure and large *S*_BET_, which have a great potential for dye adsorption.

#### 3.1.3 Surface morphology analysis

The microscope morphologies of GSBC and LSBC was observed by SEM at a magnification of ×2000 (Supplementary Figure S1). Biochars exhibited uneven fold surfaces with compact rigid structures at a pyrolysis temperature of 350°C. With the increase in temperature, the biochar surface became rough and appeared larger porous or stomata, this might be due to the decomposition of cellulose, hemicellulose, and other components during pyrolysis (Deng et al., 2022; Galgali et al., 2022). Some visible differences on surface morphology were observed in high-temperature biochars. GS750 showed a rugged flaky layered structure with small fragments on surface, while LS750 had several folds and loose holes on its flocculent structure. The hollow honeycomb-like structure and granular debris agglomerates on the surface of LS750 contributed to its larger *S*_BET_, which was in accordance with the above BET analysis (Figure 1).

#### 3.1.4 XRD analysis

**FIGURE 2:**
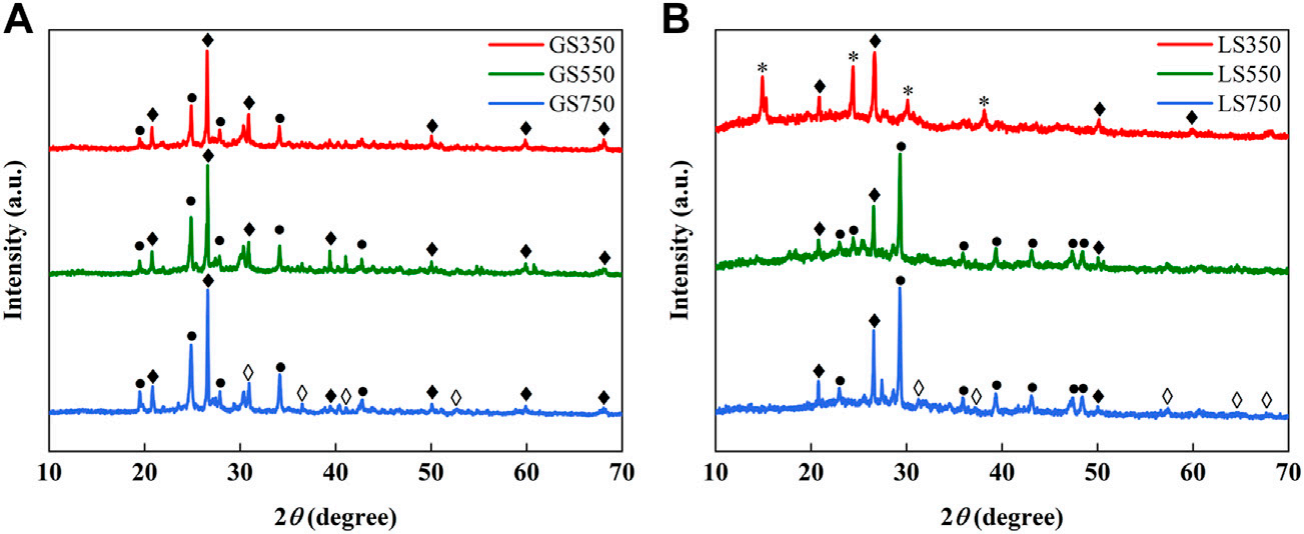
XRD patterns of GSBC **(A)** and LSBC **(B)** prepared at different pyrolysis temperature. ♦. Quartz, SiO_2_; ●. Calcite, CaCO_3_; ♦ lime, CaO; *. Calcium oxalate, CaC_2_O_4_.

The XRD analysis was studied to further investigate the mineral components in GSBC and LSBC (Figure 2). The XRD patterns showed that the crystals in biochars were mainly composed of quartz (SiO_2_) and calcite (CaCO_3_). As pyrolysis temperature increased, an enlarged peak intensity of calcite (CaCO_3_) and reduced peak intensity of quartz (SiO_2_) were observed in biochars. This was similar to the result of biochars derived from cow manure and its vermicompost (Zhang et al., 2020). Higher peak intensities of CaCO_3_ crystals observed in LSBC than GSBC further confirmed the finding that more Ca and ash inculding in LSBC (Table 1). In LS350, there appeared some peaks of calcium oxalate (CaC_2_O_4_) crystals, but they disappeared and formed new peaks of CaCO_3_ crystals in LS550 and LS750. The probable reason may be that CaC_2_O_4_ is easy to decompose into CaCO_3_ and CO at the temperature above 550 °C. As shown in Figure 2, it could be found that new peaks of CaO crystals appeared in GS750 and LS750 which might be due to a small amount of calcite decomposed to CaO and CO_2_ at around 700 °C (Karunadasa et al., 2019). Many researches have confirmed that the calcium phase (i.e., calcite and CaO), play an important role in dye sorption (Dai et al., 2018). For example, the calcium-rich adsorbent like sea shells power and crab shell derived biochar showed highly efficient adsorption of cationic dyes MG involved mechanism of electrostatic attraction (Chowdhury and Saha 2010; Dai et al., 2018). Thus, the natural minerals in carbon material should be fully preserved in the preparation of dye adsorbents.

#### 3.1.5 FTIR analysis

**FIGURE 3:**
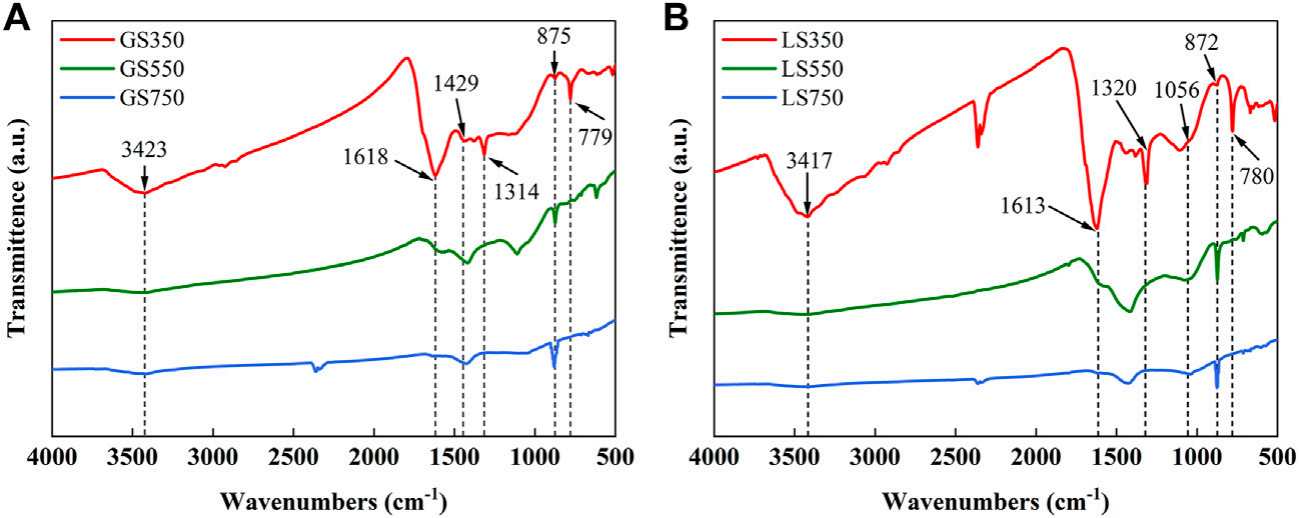
FTIR spectra of GSBC **(A)**, and LSBC **(B)** prepared at different pyrolysis temperature.

The FTIR spectra analysis was used to identify the functional groups on the surface of GSBC and LSBC (Figure 3). Peak intensities of the most functional groups on the surface of biochars declined as the rising pyrolysis temperature, while peaks near 1,436–1,429 cm^−1^ and 879–875 cm^−1^ showed the opposite trend. In specific, a broad band at 3,417–3,423 cm^−1^ represents the stretching of -OH and -NH of acidic groups and aliphatic compounds (Dai et al., 2017), which become weak or even disappeared with the rising temperature, suggesting the hydroxyl breaking of hydrogen bonds and the dehydration and decomposition of aliphatic compound occurred at high pyrolysis temperature (Bondarchuk et al., 2007; Xu et al., 2018). The band near 1,618 cm^−1^ was assigned to C=C and C=O in the aromatic ring (Xu et al., 2013; Khanday et al., 2016), and the band at 1,049–1,045 cm^−1^ represents the C-O stretching vibration of the aromatic ring (Sewu et al., 2019), and the band near 780 cm^−1^ belongs to C-H stretching in aromatic compounds (Khanday et al., 2016). At higher temperatures, these band intensities gradually declined attributed to the condensation of activated carbon atoms into the aromatic structure (Jin et al., 2018). The band at 1,314–1,320 cm^−1^ corresponded to carboxyl (-COOH) vibration (Dai et al., 2017), the weakening of the peak near 1,314 cm^−1^ was due to the decarboxylation caused by pyrolysis. The peaks near 1,436–1,429 cm^−1^ and 879–875 cm^−1^ are mainly from the C=O and C-O stretching vibration of carbonates (Dai et al., 2017). The enlarged intensity and area of peak at 1,436–1,429 cm^−1^ and 879–875 cm^−1^ implied that high temperature was beneficial to the formation of carbonate minerals (e.g. calcium salt) (Ahmad et al., 2012), which was consistent with the results of XRD analysis (Figure 2).

### 3.2 Adsorption study

#### 3.2.1 Effect of pH

**FIGURE 4:**
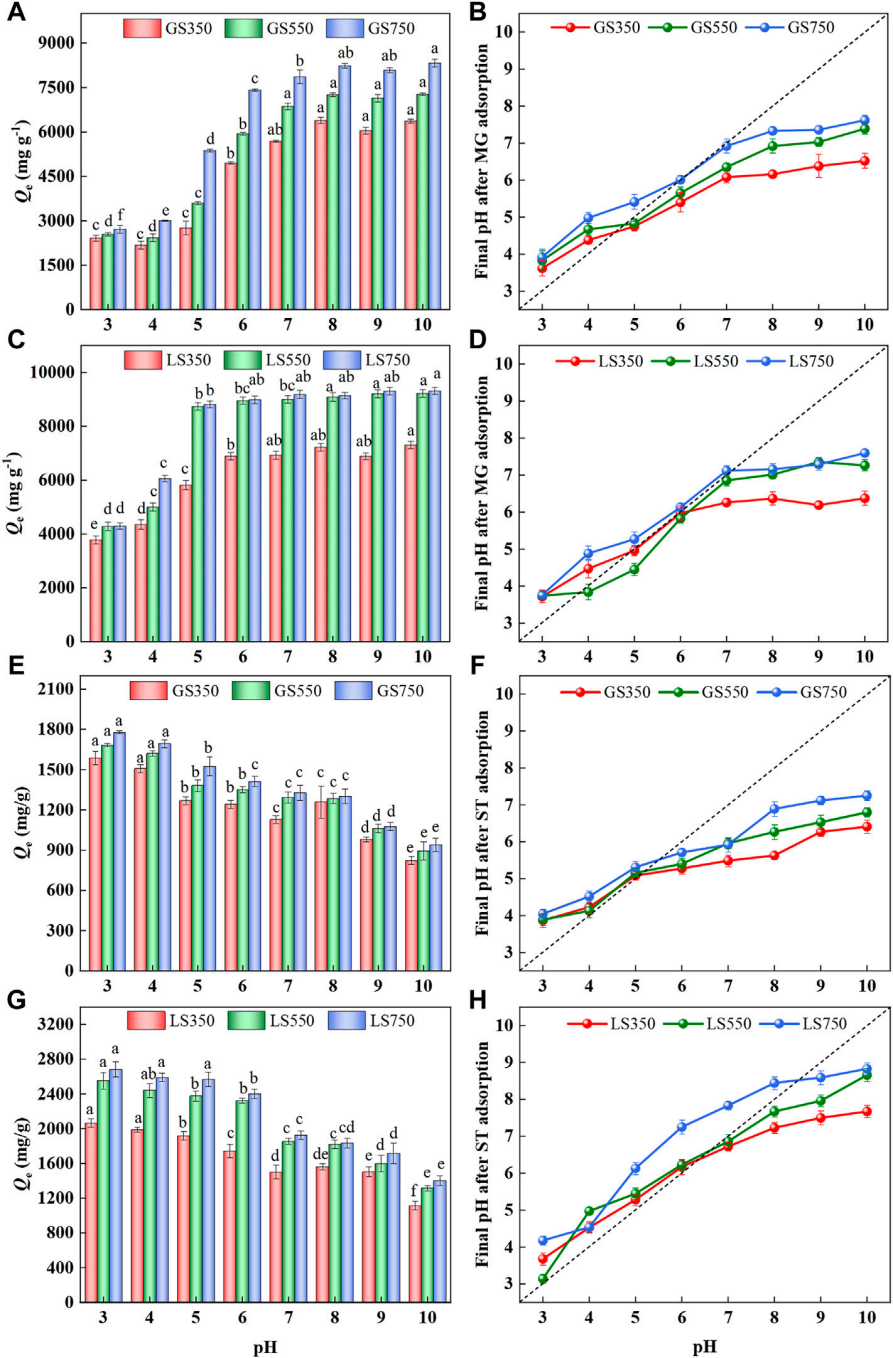
Effect of initial pH on adsorption of MG **(A,C)** and ST (e.g.,) by GSBC and LSBC, and final pH value after dye adsorption **(B,D)**, (f and h). [Conditions] For MG or ST, initial concentration of 3,500 mg L^−1^, 15 mg biochar, initial pH of 3–10, 24 h. The error bars represent the standard deviation of the mean values. The different letters above the histogram indicate significant differences at *p* < 0.05 (ANOVA and Duncan’s multiple range test using SPSS 26.0).

The solution pH affects the active sites on the adsorbent as well as the speciation of the adsorbate in the solution. The amounts of two cationic dyes (MG and ST) sorption onto biochars within the initial pH of 3.0–10.0 were studied. As shown in Figure 4, pH obviously affected two dyes’ adsorption. The adsorption of MG by two biochars increased significantly at initial pH between 3.0 and 6.0 (*p* < 0.05), then rises slowly and finally tends to a constant at pH 8 (Figures 4A,B). Cationic dye of MG was positively charged at the position containing the chromogenic groups after ionization in the aqueous solution. In acidic conditions, H^+^ and MG undergo fiercely competitive adsorption on the available sites of the biochar surface, which weakens its adsorption performance (Chowdhury and Saha 2010). On the other hand, electrostatic repulsion occurred between MG and the positively charged adsorbent surfaces due to the protonation of functional groups. As pH increased, the protonation weakened, and more available sites were provided for dye adsorption (Sewu et al., 2019). When pH > 7.0, amounts of OH^−^ are attached to the surface of biochar and it is quickly combined with positively charged MG through electrostatic attraction (Sewu et al., 2019). However, the adsorption of ST by two biochars showed an opposite trend to MG, which declined significantly with the increased pH from 4.0 to 10.0 (*p* < 0.05) (Figures 4E,G). This might be due to the solubility of ST being higher under acidic conditions, whereas ST molecules may associate with each other in alkaline solution to produce dimer or even higher molecular weight aggregates (Liu et al., 2017). Consequently, there was a certain steric hindrance effect between ST molecules and biochars, thus causing the declined adsorption capacities at alkaline pH. As for adsorption capacities, LSBC showed better performance than GSBC under the same pH condition due to it had larger *S*_BET_ and more inorganic calcium salt, especially at high temperatures.

By comparing the changes in pH before and after adsorption (Figures 4B,D,F,H), it was found that the final pH increased after dyes adsorption under acidic conditions, the possible reason is that the protonation of functional groups on biochar surface as well as the buffering effect of the inorganic elements in biochar (Su et al., 2021). When in alkaline solution, amounts of OH^−^ were combined with the surface functional groups of biochar and involved in dyes adsorption, eventually causing the declined pH. Overall, the solution pH would approach neutral after biochar adsorption, which further indicated that the biochar had a buffer effect.

#### 3.2.2 Adsorption kinetics

**FIGURE 5:**
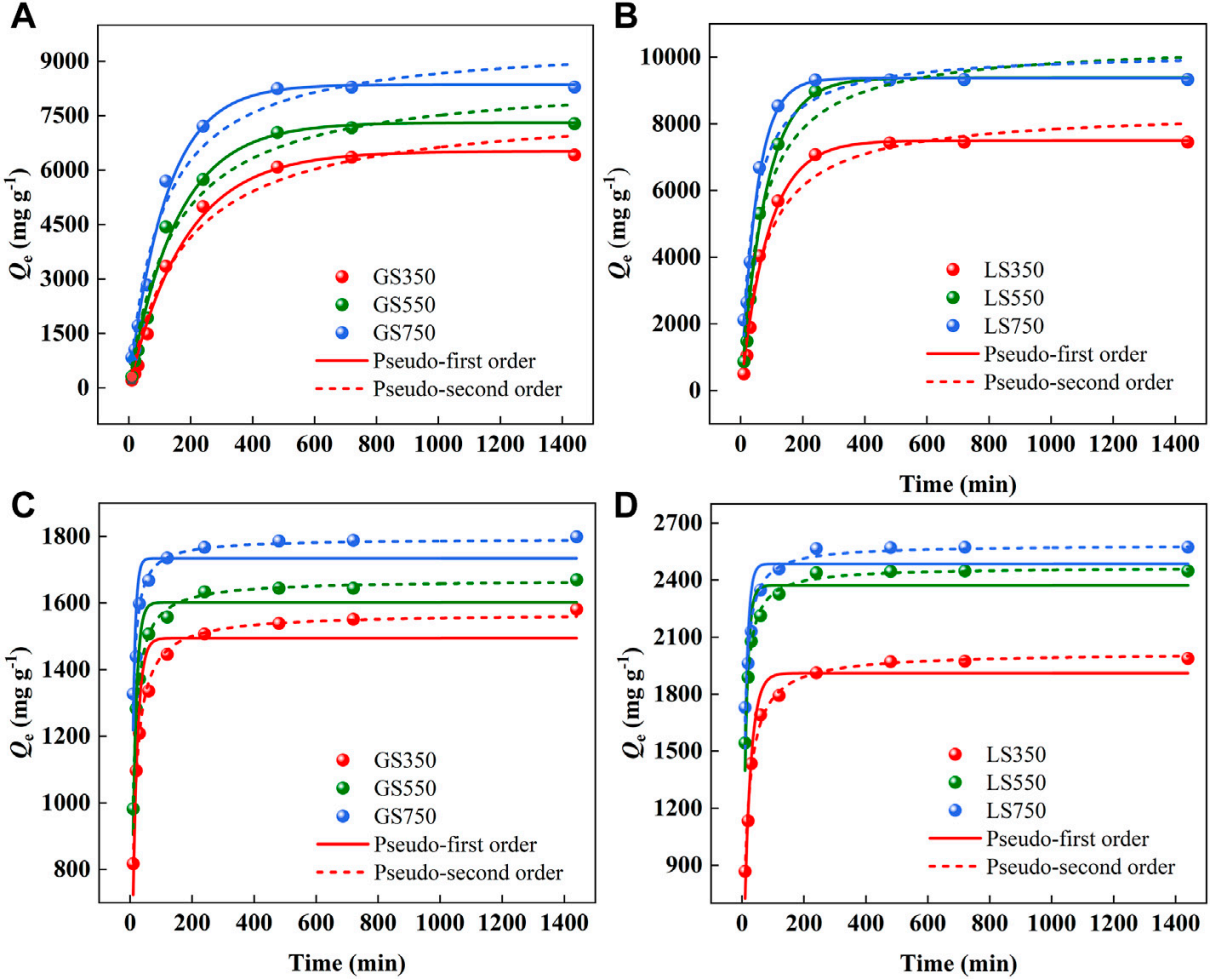
The kinetic models of MG **(A,B)** and ST **(C,D)** sorption onto GSBC and LSBC. [Conditions] For MG and ST, initial pH of 8 and 4, respectively; initial concentration of 3,500 mg L^−1^ dye, 15 mg biochar, 10–1,440 min.

The effect of contact time on the adsorption of dyes onto both biochars was shown in Figure 5. The adsorption capacities of GSBS and LSBC showed similar trends as time. In the initial adsorption stage, the adsorption rate was fast since there were sufficient active sites on the biochar surface. Then, the growing rate slowed down and tended to zero finally which due to most of the active sites on the adsorbent surface were occupied by dye molecules thus hinder them further diffusing to the internal pores (Malik 2003). The adsorption of MG and ST by LSBC was basically completed in 240 min, which was much shorter than 480 min by ASBC, probably owing to the greater *S*_BET_ and porosity of LSBC that would provide more accessible paths for adsorption. The fitting data showed that pseudo-first order (*R* ^2^ > 0.99) was the better model for describing MG sorption on GSBC and LSBC than pseudo-second order (Supplementary Table S1), indicating that diffusion is the rate controlling process (Lyu et al., 2022). While for ST, the pseudo-second order model fitted better (*R* ^2^ > 0.97), which relied on the assumption that the rate-limiting step might be chemical sorption or chemisorption involving process (Pérez-Marín et al., 2007). Compared with ST, the improved adsorption capacity of MG indicated that diffusion was the main rate-controlling step which might be more beneficial to adsorption.

**FIGURE 6:**
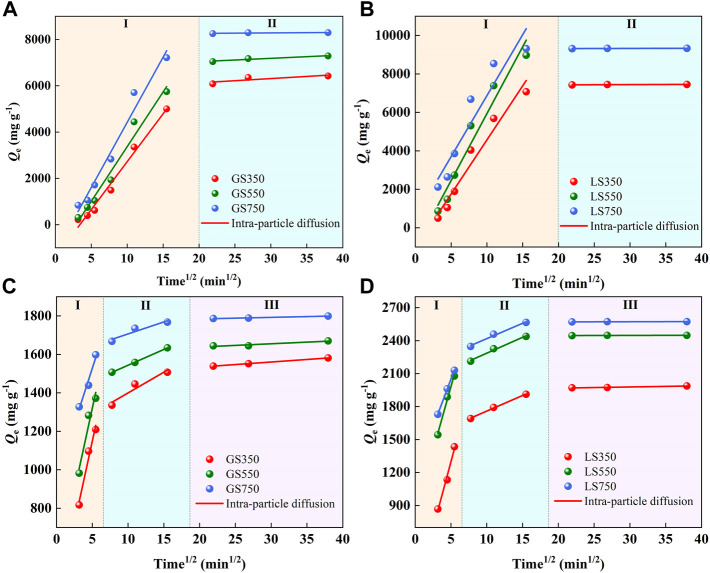
The intra-particle diffusion models of MG (a, b) and ST (c, d) sorption onto GSBC and LSBC. I, II, and III represent the different stages of intra-particle diffusion, respectively. [Conditions] For MG and ST, initial pH of 8 and 4, respectively, initial concentration of 3,500 mg L^−1^ dye, 15 mg biochar, 10–1,440 min.

The intra-particle diffusion model was used to describe the diffusion process of dyes in the adsorbent. The adsorption process can be divided into three linear stages, 1) the dye molecules diffused rapidly into the outer surface of the adsorbent, 2) the dye entered the internal pores and diffused in the pore fluid, and 3) the dye was adsorbed on the inner surface of the pores. As shown in Figure 6, MG adsorption presented two diffusion stages whereas ST adsorption showed three diffusion stages because the intraparticles diffused in pores of progressively smaller sizes up to mesopores for MG and micropores for ST (Sewu et al., 2019). All fitted lines of the intra-particle diffusion models did not pass through the origin, indicating the intra-particle diffusion was not the only rate-limiting step in dye adsorption (Dai et al., 2017). As shown in Supplementary Table S2, the changes trends of *k*_p1_>*k*_p2_>*k*_p3_ confirmed that the adsorption and diffusion rate declined as the adsorption time, which was consistent with the results of Nethaji et al. (Nethaji et al., 2018) using the waste polyurethane foam to prepare activated carbon for MG adsorption.

#### 3.2.3 Adsorption isotherm analysis

**FIGURE 7:**
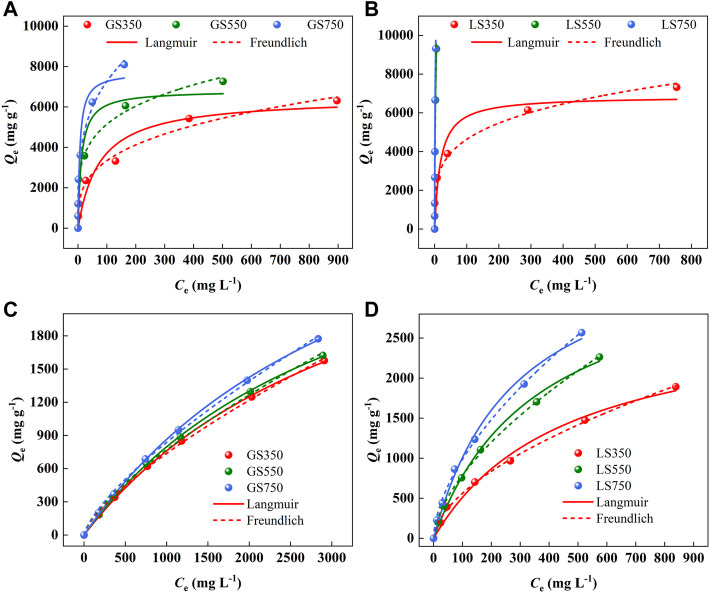
The adsorption isotherms of MG **(A,B)** and ST **(C,D)** sorption onto GSBC and LSBC. [Conditions] For MG and ST, initial pH of 8 and 4, respectively, initial concentrations of 250–3,500 mg L^−1^, 24 h.

The isotherm adsorption models were used to describe adsorption performance and the distribution of the dye at the biochar interface. As shown in Figure 7, the rapid and increased adsorption capacity occurred at low initial concentration due to more adsorption sites and active groups on the biochar surface. As the concentration increased, the adsorption sites tended to be saturated, and eventually adsorption capacity reach equilibrium. For the adsorption of MG and ST, the Freundlich model was more appropriate to describe the actual adsorption process (Figure 7 a-d, Supplementary Table S3), suggesting that the adsorption of two dyes on GSBC and LSBC was nonuniform and multilayered (Su et al., 2021). The 1/*n* value were all between 0 and 1, indicative of the favorable adsorption for dyes onto both biochars (Wang et al., 2018). The Freundlich *K*_F_ value was used to analyze the binding affinity between pollution and adsorbent in the adsorption process. *K*_F_ value increased as the rising pyrolysis temperature confirmed that high temperature was conducive to adsorption performance. It was also found that the affinity for MG was higher than that for ST which was consistent with their adsorption capacity. The maximum adsorption capacity of MG and ST to LS750 calculated by the Langmuir model were 9,388.04 and 3,871.48 mg g^−1^, respectively. Compared with the reported results (Table 2), LSBC showed a very strong adsorption capacity for both cationic dyes.

**TABLE 2:** Maximum adsorption capacity of MG and ST by various adsorbents.

Dyes	Materials	*Q*_e_ (mg g^−1^)	References
MG	Biochar from spent *Ganoderma lucidum* substrate (LS750)	9,388	This study
	Zeolitie imidazolate framework (ZIF)-sponge	4,093	Lin and Chang (2015)
	ZIF composited with graphene oxide	3,300	Abdi et al. (2017)
	Fibrous cellulose sulfate	945	Baghdadi et al. (2017)
	Nickel nanoparticles	898	Jin et al. (2018)
ST	Biochar from spent *Ganoderma lucidum* substrate (LS750)	3,871	This study
	Deoiled-mustard	3,087	Gupta et al. (2011)
	Crosslinked-acrylic acid/acrylamidopropane sulfonic acid hydrogels	2,374	Akkaya et al. (2009)
	activated carbon	635	Wang et al. (2016)
	MIL-101(Cr)-SO3H	426	Zhao et al. (2017)

#### 3.2.4 Regeneration performance analysis

**FIGURE 8:**
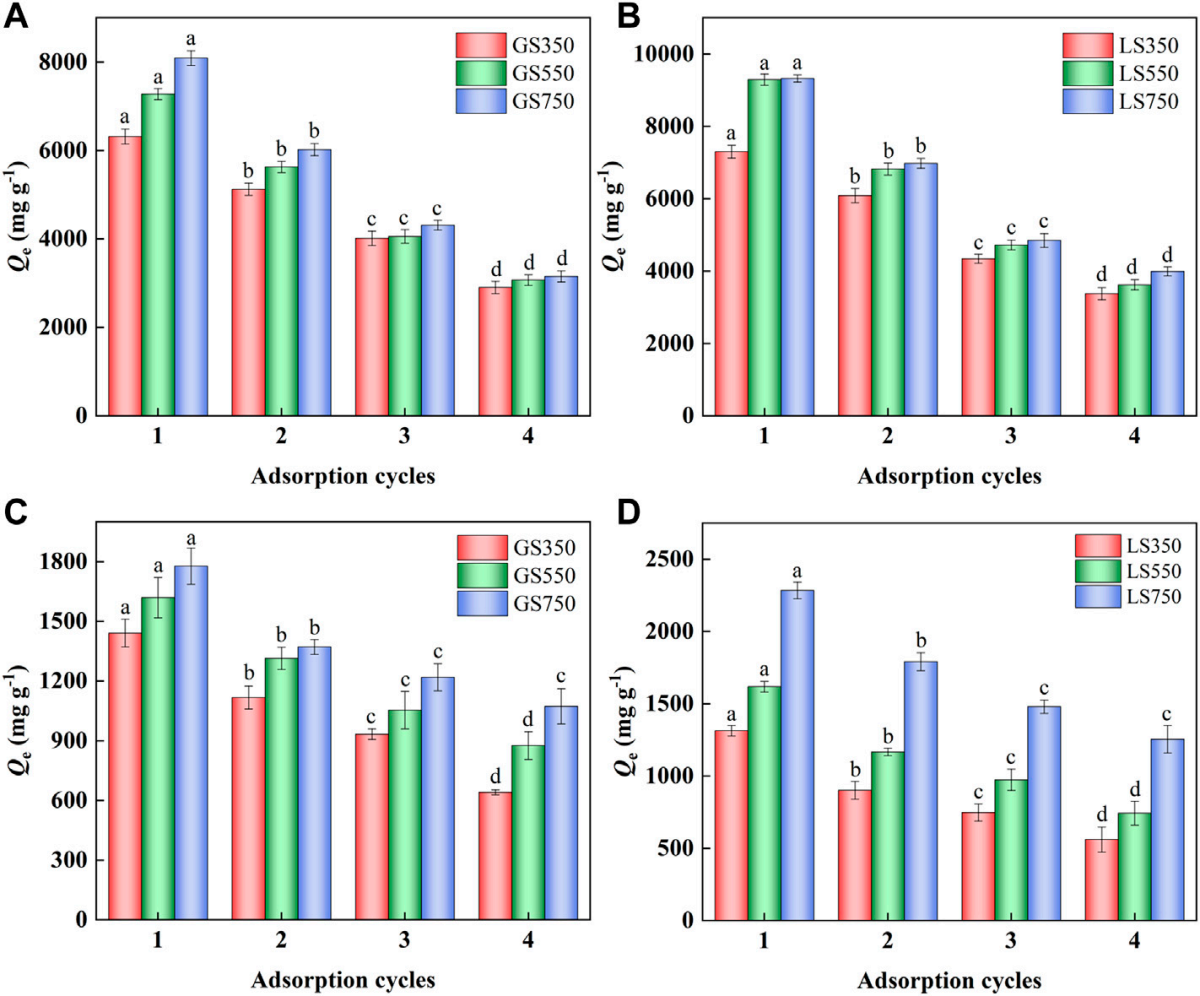
Reabsorption experiments of MG **(A,B)** and ST **(C,D)** sorption onto GSBC and LSBC. For MG and ST, initial pH of 8 and 4, respectively, initial concentration of 3,500 mg L^−1^, 24 h. The error bars represent the standard deviation of the mean values. The different letters above the histogram indicate significant differences at *p* < 0.05 (ANOVA and Duncan’s multiple range test using SPSS 26.0).

The recyclability of an adsorbent is very important in practical applications. Dyes adsorbed on GSBC and LSBC were desorbed by the ethanol washing method, and the re-adsorption performance was analyzed. As shown in Figure 8, the adsorption performance of GSBC and LSBC significantly became weak as the increased adsorption cycles (*p* < 0.05). Although ethanol washing recover partial available active sites of dye-adsorbed biochar, the formed inert compounds enter the biochar pores, and the larger volume became with the increase of adsorption cycles. These compounds could not escape from the biochar pore thereby resulting in low dye adsorption performance. In addition, continuous adsorption-desorption would cause the abrasion of the adsorption site and further destroy the surface morphology of adsorbent. However, it was clear that the adsorption capacities of GS750 and LS750 were still as high as >3,000 mg g^−1^ for MG and >1,000 mg g^−1^ for ST after four cycles. Thus, even though the regenerated biochars showed lower adsorption performance than the primary ones, they can still be recognized as superior adsorbents compared with other adsorbents (Table 2).

#### 3.2.5 Adsorption mechanisms

The analysis of the dye adsorption mechanism is of significance to evaluate the application potential of biochars and their impact on the environment. According to the results of BET (Figure 1), GSBC and LSBC were the mesoporous materials (average pore size: 2–50 nm). In general, the smaller average pore size, the larger specific surface area, and the stronger adsorption capacity (Cai et al., 2013). High-temperature biochars (GS750 and LS750) had a larger specific surface area and a stronger adsorption capacity, indicating that more adsorption sites were provided for dye molecules. Thus, the specific surface adsorption or pore filling was one of the possible reasons for good adsorption capacities of these biochars.

As shown in FTIR spectra (Supplementary Figure S2), peaks at 1,314–1,320 cm^−1^, 1,049–1,082 cm^−1^, 779 cm^−1^ assigned to carboxyl group vibration (Dai et al., 2017), aromatic C-O vibration and aromatic C-H vibration (Sewu et al., 2019), respectively, have distinct differences after dye adsorption. This further confirmed the MG and ST successfully adsorbed onto the biochars. The absorption bands of GSBC and LSBC representing O-H or N-H at 3,417–3,423 cm^−1^ changed obviously after MG adsorption, which indicated that the hydrogen bonding force involved in MG adsorption (Du et al., 2014), whereas no obvious change could be found after ST adsorption, implying that the role of hydrogen bonding was very weak in ST adsorption. The bands at 1,613–1,618 cm^−1^ changed significantly, which probably because the π-π stacking interaction between the aromatic π electrons on biochar and benzene ring structure of the dye (Wu et al., 2014). The negatively charged functional groups like the carboxyl group (-COO^-^) at 1,049–1,082 cm^−1^, hydroxyl group (-O^-^) at 3,417–3,423 cm^−1^, and CO_3_^2-^ at 875–879 cm^−1^, might be combined with the positively charged chromogenic group N^+^ in MG and ST by electrostatic attraction. Besides, Table1 showed that SMSs derived biochars (especially high-temperature biochars) were rich in Ca^2+^ with a small number of metal ions (Mg^2+^, K^+^, and Na^+^) concurrently. Thus, ion exchange may also exist in the adsorption process of the MG and ST (Park et al., 2019).

The typical bands for calcite (CaCO_3_) at 1,429–1,436 cm^−1^ and 875–879 cm^−1^ changed significantly after dyes adsorption, indicating that calcite in biochar participated in the dye adsorption process (Dai et al., 2018). For instance, the anion of CO_3_^2-^ in carbonate minerals adsorbed cationic dye through electrostatic attraction (Chowdhury and Saha 2010; Zhang et al., 2020). Moreover, many studies have found that mineral phases such as calcite and CaO in biochars are involved in dye adsorption (Chowdhury and Saha 2010). According to the analysis of XRD (Figure 2), SMSs derived biochars were rich in calcite and CaO, especially at high temperature condition (GS750 and LS750), this might be another reason for their high adsorption capacities of dyes compared with other adsorbents (Table 2).

**FIGURE 9:**
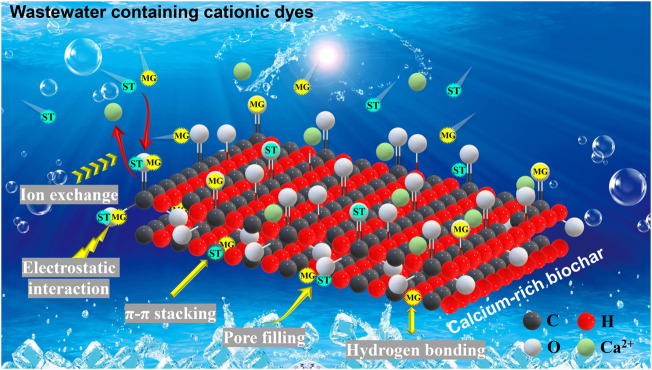
Proposed a possible adsorption mechanism of the cationic dyes from wastewater by the calcium-rich biochar derived from spent mushroom substrates.

Therefore, the role of calcium minerals in SMS-biochars in adsorbing dye pollutants should not be overlooked. In fact, many studies have confirmed that carbon materials containing calcium minerals are effective for dye adsorption (Chowdhury and Saha 2010; Dai et al., 2018; Sahithya et al., 2022). Based on the above findings, we conclude that the calcium minerals in SMS-biochars mainly consist of calcite and CaO, which participate in the dye adsorption by two possible mechanisms, ion exchange (Ca^2+^) and electrostatic attraction (CO_3_^2-^). LSBC had higher calcium content (Table 1) and larger *S*_BET_ (Figure 1) than that of GSBC at all pyrolysis temperature, thereby it owned excellent adsorption capacity for both cationic dyes (MG and ST). Furthermore, based on the above results, it was found that pore filling, hydrogen bonding, electrostatic interaction, ion exchange, and π-π stacking were involved in the adsorption of MG onto SMS-biochars, while ST adsorption mainly involved pore filling, electrostatic interaction, ion exchange, and π-π stacking (Fig. 9). This might cause the distinct performance in the adsorption of MG and ST by SMS-biochars. Overall, a possible mechanism was proposed in this study to explain the adsorption superiority of calcium-rich bochars from SMSs, which benefits the removal of cationic dyes from wastewater.

#### 3.2.6 Practical implications

The biochar-inherent minerals played significant role on its sorption properties and applications (Xu et al., 2017). SMS typically consists of both carbon and mineral fractions, and minerals including high level of calcium, which were mainly from the biomass itself and/or artificial additives. Consequently, calcium-rich biochars can be produced via the direct pyrolysis of SMSs. Currently, SMS based biochar for the removal of pollutants from wastewater has captured much scholarly attention due to its highly efficient, low-cost, and eco-friendly (Sewu et al., 2019; Chang et al., 2020). This work concluded that calcium-rich biochars from SMSs (GSBC and LSBC) showed excellent adsorption performance in removing cationic dyes (MG and ST) in water. Therefore, it has broad application prospects in the removal of cationic dyes as represented by MG and ST. Unfortunately, the adsorption of anionic dyes was not satisfactory according to the results of preliminary experiment. Given the extensive application of SMS-biochars, modification methods of SMS-biochars still need to be well designed that minimize any potential loss of minerals. Especially, the effective regulation of surface functional groups on SMS-biochars is highly needed to adsorb both cationic and anionic dyes. Our work has explained several mechanisms involved in dye adsorption, however, it is still unclear which mechanism plays a key role and how much it contributes. Moreover, for better understand the adsorption mechanism, our research primarily focused on the removal of dyes from a single-adsorptive system, more work should be done on the adsorption mechanism in multiple-adsorptive system. Clarifying the above issues is incredibly important for improving the adsorption performance and environmental applications of SMS-biochars, and it is also the key step in the future industrialization applications.

## 4 Conclusion

Calcium-rich biochars (GSBC and LSBC) were directly pyrolyzed from two types of spent substrates of *Ganoderma lucidum* and *Lentinus edodes* without any modification. High pyrolysis temperature led to an increase in both biochar ash, Ca content, and specific surface area, which made high-temperature biochars (GS750 and LS750) the superior adsorbents for cationic dyes (MG and ST). Batch adsorption results showed LS750 was more efficient to adsorb dyes than GS750 because its higher Ca content and larger specific surface area. According to the Langmuir model, LS750 had high adsorption capacities of 9,388.04 and 3,871.48 mg g^−1^ for MG and ST, respectively. The adsorption mechanism of MG could be attributed to pore filling, hydrogen bonding, electrostatic interaction, ion exchange, and π-π stacking, while ST adsorption mainly involved pore filling, electrostatic interaction, ion exchange, and π-π stacking. Due to its excellent adsorption performance, cheap source, good reusability, biochars obtained from SMSs were very promising in dyeing wastewater treatment.

## Data Availability

The raw data supporting the conclusions of this article will be made available by the authors, without undue reservation.

## References

[B1] Abdel-Khalek M. Rahman M. A. Francis A. (2017). Exploring the adsorption behavior of cationic and anionic dyes on industrial waste shells of egg. J. Environ. Chem. Eng. 5 (1), 319–327. 10.1016/j.jece.2016.11.043

[B2] Abdi J. Vossoughi M. Mahmoodi N. M. Alemzadeh I. (2017). Synthesis of metal-organic framework hybrid nanocomposites based on GO and CNT with high adsorption capacity for dye removal. Chem. Eng. J. 326, 1145–1158. 10.1016/j.cej.2017.06.054

[B3] Ahmad M. Lee S. S. Dou X. Mohan D. Sung J.-K. Yang J. E. (2012). Effects of pyrolysis temperature on soybean stover-and peanut shell-derived biochar properties and TCE adsorption in water. Bioresour. Technol. 118, 536–544. 10.1016/j.biortech.2012.05.042 22721877

[B4] Akkaya M. Ç. Emik S. Güçlü G. İyim T. B. Özgümüş S. (2009). Removal of basic dyes from aqueous solutions by crosslinked‐acrylic acid/acrylamidopropane sulfonic acid hydrogels. J. Appl. Polym. Sci. 114 (2), 1150–1159. 10.1002/app.30704

[B5] Anastopoulos I. Mittal A. Usman M. Mittal J. Yu G. Núñez-Delgado A. (2018). A review on halloysite-based adsorbents to remove pollutants in water and wastewater. J. Mol. Liq. 269, 855–868. 10.1016/j.molliq.2018.08.104

[B6] Arora C. Kumar P. Soni S. Mittal J. Mittal A. Singh B. (2020). Efficient removal of malachite green dye from aqueous solution using Curcuma caesia based activated carbon. Desalination Water Treat. 195, 341–352. 10.5004/dwt.2020.25897

[B7] Azari A. Nabizadeh R. Nasseri S. Mahvi A. H. Mesdaghinia A. R. (2020). Comprehensive systematic review and meta-analysis of dyes adsorption by carbon-based adsorbent materials: Classification and analysis of last decade studies. Chemosphere 250, 126238–126318. 10.1016/j.chemosphere.2020.126238 32092572

[B8] Baghdadi M. Soltani B. A. Nourani M. (2017). Malachite green removal from aqueous solutions using fibrous cellulose sulfate prepared from medical cotton waste: Comprehensive batch and column studies. J. Ind. Eng. Chem. 55, 128–139. 10.1016/j.jiec.2017.06.037

[B9] Bondarchuk O. Kim Y. K. White J. Kim J. Kay B. D. Dohnalek Z. (2007). Surface chemistry of 2-propanol on TiO2 (110): Low-and high-temperature dehydration, isotope effects, and influence of local surface structure. J. Phys. Chem. C 111 (29), 11059–11067. 10.1021/jp072298m

[B10] Buss W. Graham M. C. Shepherd J. G. Mašek O. (2016). Suitability of marginal biomass-derived biochars for soil amendment. Sci. Total Environ. 547, 314–322. 10.1016/j.scitotenv.2015.11.148 26789369

[B11] Cai Y. D. Liu D. M. Pan Z. J. Yao Y. B. Li J. Q. Qiu Y. K. (2013). Pore structure and its impact on CH_4_ adsorption capacity and flow capability of bituminous and subbituminous coals from Northeast China. Fuel 103, 258–268. 10.1016/j.fuel.2012.06.055

[B12] Chang J. N. Zhang H. B. Cheng H. Y. Yan Y. Y. Chang M. C. Cao Y. Z. (2020). Spent Ganoderma lucidum substrate derived biochar as a new bio-adsorbent for Pb^2+^/Cd^2+^ removal in water. Chemosphere 241, 125121. 10.1016/j.chemosphere.2019.125121 31683424

[B13] Choudhary M. Kumar R. Neogi S. (2020). Activated biochar derived from Opuntia ficus-indica for the efficient adsorption of malachite green dye, Cu^2+^ and Ni^2+^ from water. J. Hazard. Mat. 392, 122441. 10.1016/j.jhazmat.2020.122441 32193109

[B14] Chowdhury S. Saha P. (2010). Sea shell powder as a new adsorbent to remove Basic Green 4 (Malachite Green) from aqueous solutions: Equilibrium, kinetic and thermodynamic studies. Chem. Eng. J. 164 (1), 168–177. 10.1016/j.cej.2010.08.050

[B15] Dai L. C. Tan F. R. Li H. Zhu N. M. He M. X. Zhu Q. L. (2017). Calcium-rich biochar from the pyrolysis of crab shell for phosphorus removal. J. Environ. Manage. 198 (1), 70–74. 10.1016/j.jenvman.2017.04.057 28453987

[B16] Dai L. C. Zhu W. K. He L. Tan F. R. Zhu N. M. Zhou Q. (2018). Calcium-rich biochar from crab shell: an unexpected super adsorbent for dye removal. Bioresour. Technol. 267, 510–516. 10.1016/j.biortech.2018.07.090 30048926

[B17] Deng P. Y. Wan W. X. Azeem M. Riaz L. Zhang W. Yang Y. Y. (2022). Characterization of biochar derived from bamboo and its application to modulate the toxic effects of chromium on wheat plant. Biomass Convers. biorefin.. 10.1007/s13399-022-02879-2

[B18] Du Q. J. Sun J. K. Li Y. H. Yang X. X. Wang X. H. Wang Z. H. (2014). Highly enhanced adsorption of congo red onto graphene oxide/chitosan fibers by wet-chemical etching off silica nanoparticles. Chem. Eng. J. 245, 99–106. 10.1016/j.cej.2014.02.006

[B19] Dutta S. Gupta B. Srivastava S. K. Gupta A. K. (2021). Recent advances on the removal of dyes from wastewater using various adsorbents: A critical review. Mat. Adv. 2 (14), 4497–4531. 10.1039/D1MA00354B

[B20] Galgali P. Palimkar S. Adhikar A. Patel R. Routh J. (2022). Remediation of potentially toxic elements-containing wastewaters using water hyacinth–a review. Int. J. Phytoremediation. 10.1080/15226514.2022.2068501 35522852

[B21] Gao X. H. Tang X. Y. Zhao K. Y. Balan V. Zhu Q. L. (2021). Biogas production from anaerobic Co-digestion of spent mushroom substrate with different livestock manure. Energies 14 (3), 570. 10.3390/en14030570

[B22] Gupta V. K. Jain R. Saleh T. Nayak A. Malathi S. Agarwal S. (2011). Equilibrium and thermodynamic studies on the removal and recovery of safranine-T dye from industrial effluents. Sep. Sci. Technol. 46 (5), 839–846. 10.1080/01496395.2010.535591

[B23] Gurbani D. Kukshal V. Laubenthal J. Kumar A. Pandey A. Tripathi S. (2012). Mechanism of inhibition of the ATPase domain of human topoisomerase IIα by 1, 4-benzoquinone, 1, 2-naphthoquinone, 1, 4-naphthoquinone, and 9, 10-phenanthroquinone. Toxicol. Sci. 126 (2), 372–390. 10.1093/toxsci/kfr345 22218491

[B24] Han H. W. Rafiq M. K. Zhou T. Y. Xu R. Mašek O. Li X. K. (2019). A critical review of clay-based composites with enhanced adsorption performance for metal and organic pollutants. J. Hazard. Mat. 369, 780–796. 10.1016/j.jhazmat.2019.02.003 30851518

[B25] Hokkanen S. Bhatnagar A. Sillanpää M. (2016). A review on modification methods to cellulose-based adsorbents to improve adsorption capacity. Water Res. 91, 156–173. 10.1016/j.watres.2016.01.008 26789698

[B26] Hu S. X. Hsieh Y. L. (2017). Lignin derived activated carbon particulates as an electric supercapacitor: carbonization and activation on porous structures and microstructures. RSC Adv. 7 (48), 30459–30468. 10.1039/C7RA00103G

[B27] Jin L. N. Zhao X. S. Qian X. Y. Dong M. D. (2018). Nickel nanoparticles encapsulated in porous carbon and carbon nanotube hybrids from bimetallic metal-organic-frameworks for highly efficient adsorption of dyes. J. Colloid Interface Sci. 509, 245–253. 10.1016/j.jcis.2017.09.002 28915482

[B28] Karunadasa K. S. Manoratne C. Pitawala H. Rajapakse R. (2019). Thermal decomposition of calcium carbonate (calcite polymorph) as examined by *in-situ* high-temperature X-ray powder diffraction. J. Phys. Chem. Solids 134, 21–28. 10.1016/j.jpcs.2019.05.023

[B29] Khanday W. A. Kabir G. Hameed B. (2016). Catalytic pyrolysis of oil palm mesocarp fibre on a zeolite derived from low-cost oil palm ash. Energy Convers. Manag. 127, 265–272. 10.1016/j.enconman.2016.08.093

[B30] Kwak J. H. Islam M. S. Wang S. Messele S. A. Naeth M. A. El-Din M. G. (2019). Biochar properties and lead(II) adsorption capacity depend on feedstock type, pyrolysis temperature, and steam activation. Chemosphere 231, 393–404. 10.1016/j.chemosphere.2019.05.128 31146131

[B31] Lin K. Y. A. Chang H. A. (2015). A zeolitic imidazole framework (ZIF)–sponge composite prepared via a surfactant-assisted dip-coating method. J. Mat. Chem. A 3 (40), 20060–20064. 10.1039/c5ta04427h

[B32] Liu J. Shi J. H. Qian C. X. Zhao Y. B. Chen L. H. Huang L. L. (2017). Decolorization of rhodamine-B from aqueous solutions by spent mushroom substrate. BioResources 12 (4), 8612–8628. 10.15376/biores.12.4.8612-8628

[B33] Lyu W. Li J. Q. Trchová M. Wang G. Liao Y. Z. Bober P. (2022). Fabrication of polyaniline/poly (vinyl alcohol)/montmorillonite hybrid aerogels toward efficient adsorption of organic dye pollutants. J. Hazard. Mat. 435, 129004. 10.1016/j.jhazmat.2022.129004 35500341

[B34] Malik P. K. (2003). Use of activated carbons prepared from sawdust and rice-husk for adsorption of acid dyes: a case study of acid yellow 36. Dyes pigments 56 (3), 239–249. 10.1016/S0143-7208(02)00159-6

[B35] Medeiros D. C. C. d. S. Nzediegwu C. Benally C. Messele S. A. Kwak J. H. Naeth M. A. (2021). Pristine and engineered biochar for the removal of contaminants co-existing in several types of industrial wastewaters: A critical review. Sci. Total Environ. 809, 151120. 10.1016/j.scitotenv.2021.151120 34756904

[B36] Mustafa H. M. Hayder G. (2021). Recent studies on applications of aquatic weed plants in phytoremediation of wastewater: A review article. Ain Shams Eng. J. 12 (1), 355–365. 10.1016/j.asej.2020.05.009

[B37] Nethaji S. Tamilarasan G. Neehar P. Sivasamy A. (2018). Visible light photocatalytic activities of BiOBr-activated carbon (derived from waste polyurethane) composites by hydrothermal process. J. Environ. Chem. Eng. 6 (3), 3735–3744. 10.1016/j.jece.2017.02.037

[B38] Park J. H. Wang J. J. Meng Y. Wei Z. DeLaune R. D. Seo D. C. (2019). Adsorption/desorption behavior of cationic and anionic dyes by biochars prepared at normal and high pyrolysis temperatures. Colloids Surfaces A Physicochem. Eng. Aspects 572, 274–282. 10.1016/j.colsurfa.2019.04.029

[B39] Patra B. R. Mukherjee A. Nanda S. Dalai A. K. (2021). Biochar production, activation and adsorptive applications: a review. Environ. Chem. Lett. 19 (3), 2237–2259. 10.1007/s10311-020-01165-9

[B40] Pérez-Marín A. Zapata V. M. Ortuno J. Aguilar M. Sáez J. Lloréns M. (2007). Removal of cadmium from aqueous solutions by adsorption onto orange waste. J. Hazard. Mat. 139 (1), 122–131. 10.1016/j.jhazmat.2006.06.008 16846686

[B41] Ravindiran G. Ganapathy G. P. Josephraj J. Alagumalai A. (2019). A critical insight into biomass derived biosorbent for bioremediation of dyes. ChemistrySelect 4 (33), 9762–9775. 10.1002/slct.201902127

[B42] Sahithya K. Mouli T. Biswas A. T M. S. (2022). Remediation potential of mushrooms and their spent substrate against environmental contaminants: An overview. Biocatal. Agric. Biotechnol. 42, 102323. 10.1016/j.bcab.2022.102323

[B43] Sajjadi M. Ahmadpoor F. Nasrollahzadeh M. Ghafuri H. (2021). Lignin-derived (nano)materials for environmental pollution remediation: current challenges and future perspectives. Int. J. Biol. Macromol. 178, 394–423. 10.1016/j.ijbiomac.2021.02.165 33636266

[B44] Sartape A. S. Mandhare A. M. Jadhav V. V. Raut P. D. Anuse M. A. Kolekar S. S. (2017). Removal of malachite green dye from aqueous solution with adsorption technique using Limonia acidissima (wood apple) shell as low cost adsorbent. Arab. J. Chem. 10, S3229–S3238. 10.1016/j.arabjc.2013.12.019

[B45] Sewu D. D. Jung H. Kim S. S. Lee D. S. Woo S. H. (2019). Decolorization of cationic and anionic dye-laden wastewater by steam-activated biochar produced at an industrial-scale from spent mushroom substrate. Bioresour. Technol. 277, 77–86. 10.1016/j.biortech.2019.01.034 30660064

[B46] Singh R. Bokka S. Lakshya A. K. Chowdhury A. (2022). CaO-doped tetragonal ZrO_2_ nanoparticles as an effective adsorbent for the removal of organic dye waste. Appl. Surf. Sci. 596 (15), 153651. 10.1016/j.apsusc.2022.153651

[B47] Su L. Zhang H. B. Oh K. Liu N. Luo Y. Cheng H. Y. (2021). Activated biochar derived from spent Auricularia auricula substrate for the efficient adsorption of cationic azo dyes from single and binary adsorptive systems. Water Sci. Technol. 84 (1), 101–121. 10.2166/wst.2021.222 34280158

[B48] Sun Y. N. Gao B. Yao Y. Fang J. N. Zhang M. Zhou Y. M. (2014). Effects of feedstock type, production method, and pyrolysis temperature on biochar and hydrochar properties. Chem. Eng. J. 240, 574–578. 10.1016/j.cej.2013.10.081

[B49] Sutar S. Patil P. Jadhav J. (2022). Recent advances in biochar technology for textile dyes wastewater remediation: A review. Environ. Res. 209, 112841. 10.1016/j.envres.2022.112841 35120893

[B50] Tang Y. Alam M. S. Konhauser K. O. Alessi D. S. Xu S. Tian W. (2019). Influence of pyrolysis temperature on production of digested sludge biochar and its application for ammonium removal from municipal wastewater. J. Clean. Prod. 209, 927–936. 10.1016/j.jclepro.2018.10.268

[B51] Tsai C. Lin P. Y. Hsieh S. L. Kirankumar R. Patel A. K. Singhania R. R. (2022). Engineered mesoporous biochar derived from rice husk for efficient removal of malachite green from wastewaters. Bioresour. Technol. 347, 126749. 10.1016/j.biortech.2022.126749 35066130

[B52] Ugraskan V. Isik B. Yazici O. Cakar F. (2022). Removal of Safranine T by a highly efficient adsorbent (Cotinus Coggygria leaves): Isotherms, kinetics, thermodynamics, and surface properties. Surfaces Interfaces 28, 101615. 10.1016/j.surfin.2021.101615

[B53] Wang C. Q. Wang H. Cao Y. J. (2018). Pb (II) sorption by biochar derived from Cinnamomum camphora and its improvement with ultrasound-assisted alkali activation. Colloids Surfaces A Physicochem. Eng. Aspects 556, 177–184. 10.1016/j.colsurfa.2018.08.036

[B54] Wang K. K. Li C. F. Liang Y. X. Han T. T. Huang H. L. Yang Q. Y. (2016). Rational construction of defects in a metal-organic framework for highly efficient adsorption and separation of dyes. Chem. Eng. J. 289, 486–493. 10.1016/j.cej.2016.01.019

[B55] Wu J. Yang J. W. Feng P. Wen L. S. Huang G, H. Xu C. H. (2022). Highly efficient and ultra-rapid adsorption of malachite green by recyclable crab shell biochar. J. Ind. Eng. Chem. 113 (25), 206–214. 10.1016/j.jiec.2022.05.047

[B56] Wu Z. B. Zhong H. Yuan X. Z. Wang H. Wang L. L. Chen X. H. (2014). Adsorptive removal of methylene blue by rhamnolipid-functionalized graphene oxide from wastewater. Water Res. 67, 330–344. 10.1016/j.watres.2014.09.026 25314573

[B57] Xu X. Y. Cao X. D. Zhao L. (2013). Comparison of rice husk-and dairy manure-derived biochars for simultaneously removing heavy metals from aqueous solutions: role of mineral components in biochars. Chemosphere 92 (8), 955–961. 10.1016/j.chemosphere.2013.03.009 23591132

[B58] Xu X. Y. Zhao Y. H. Sima J. K. Zhao L. Mašek O. Cao X. D. (2017). Indispensable role of biochar-inherent mineral constituents in its environmental applications: A review. Bioresour. Technol. 241 (1), 887–899. 10.1016/j.biortech.2017.06.023 28629105

[B59] Xu Z. X. Xu L. Cheng J. H. He Z. X. Wang Q. Hu X. (2018). Investigation of pathways for transformation of N-heterocycle compounds during sewage sludge pyrolysis process. Fuel Process. Technol. 182, 37–44. 10.1016/j.fuproc.2018.10.020

[B60] Yar A. Parlayici S. (2022). Carbon nanotubes/polyacrylonitrile composite nanofiber mats for highly efficient dye adsorption. Colloids Surfaces A Physicochem. Eng. Aspects 651, 129703. 10.1016/j.colsurfa.2022.129703

[B61] Yuan J. H. Xu R. K. Zhang H. (2011). The forms of alkalis in the biochar produced from crop residues at different temperatures. Bioresour. Technol. 102 (3), 3488–3497. 10.1016/j.biortech.2010.11.018 21112777

[B62] Zhang W. W. Du W. H. Wang F. Xu H. T. Zhao T. H. Zhang H. J. (2020). Comparative study on Pb^2+^ removal from aqueous solutions using biochars derived from cow manure and its vermicompost. Sci. Total Environ. 716, 137108. 10.1016/j.scitotenv.2020.137108 32059306

[B63] Zhao W. T. Cui Y. D. Zhou S. Z. Ye J. Q. Sun J. Liu X. M. (2022). Rapid adsorption of dyes from aqueous solutions by modified lignin derived superparamagnetic composites. J. Mol. Struct. 1261 (5), 132954. 10.1016/j.molstruc.2022.132954

[B64] Zhao X. D. Wang K. K. Gao Z. Q. Gao H. H. Xie Z. X. Du X. Y. (2017). Reversing the dye adsorption and separation performance of metal–organic frameworks via introduction of− SO_3_H groups. Ind. Eng. Chem. Res. 56 (15), 4496–4501. 10.1021/acs.iecr.7b00128

[B65] Zhu L. Shen D. Luo K. H. (2020). A critical review on VOCs adsorption by different porous materials: Species, mechanisms and modification methods. J. Hazard. Mat. 389, 122102. 10.1016/j.jhazmat.2020.122102 32058893

