# Supplementary material for: A new type of calcium-rich biochars derived from spent mushroom substrates and their efficient adsorption properties for cationic dyes
Source: Front Bioeng Biotechnol. 2022 Sep 20;10:1007630. doi: 10.3389/fbioe.2022.1007630 (PMC9530940; doi:10.3389/fbioe.2022.1007630)
Supplement: Supplementary file 1 [file DataSheet1.docx]

(Total 2 texts, 2 figures and 3 tables)

**Contents**

**Text S1.** Adsorption Kinetic models.

**Text S2.** Adsorption Isotherm models.

**Fig. S1.** SEM images of GSBC and LSBC.

**Fig. S2.** FTIR spectra of GSBC and LSBC before and after adsorption of MG and ST.

**Table S1.** Parameters of pseudo-first order and pseudo-second order kinetic models of MG and ST.

**Table S2.** Parameters of intra-particle diffusion models of MG and ST.

**Table S3.** Parameters of adsorption isotherm models of MG and ST.

**Text S1. Adsorption Kinetic models.**

The kinetics of MG and ST sorption were fitted to the pseudo‐first order (Eq.1), pseudo‐second order (Eq.2), and Intra-particle diffusion models (Eq.3).

| $\text{Q}_{\text{t}}\text{=}\text{Q}_{\text{e}}\left( \text{1-}\text{e}^{{\text{-}\text{k}}_{\text{1}}\text{t}} \right)$ | (1) |
| --- | --- |
| $\text{Q}_{\text{t}}\text{=}\frac{\text{k}_{\text{2}}\text{Q}_{\text{e}}^{\text{2}}\text{t}}{\text{1+}\text{k}_{\text{2}}\text{Q}_{\text{e}}\text{t}}$ | (2) |
| $\text{Q}_{\text{t}}\text{=}\text{k}_{\text{pi}}\text{t}^{\frac{\text{1}}{\text{2}}}\text{+}\text{c}$ | (3) |

where $\text{Q}_{\text{t}}$ is the sorbate adsorbed at time t, (mg g^-1^); $\text{Q}_{\text{e}}$ is the adsorption capacity of adsorbent at equilibrium, (mg g^-1^); t is the adsorption time, (min); $\text{k}_{1}$ is the pseudo-first order rate constant, (min^-1^); $\text{k}_{2}$ is the pseudo-second order rate constant, (min^-1^); $\text{k}_{\text{pi}}$ is intraparticle diffusion rate constant (mg g^-1^min^-1/2^); $\text{c}$ is the boundary layer constant.

**Text S2. Adsorption Isotherm models**

The Langmuir isotherm model (Eq.4) and Freundlich isotherm model (Eq.6) were used to simulate MG and ST sorption to biochars.

| $\text{Q}_{\text{e}}\text{=}\frac{\text{Q}_{\text{max}}\text{K}_{\text{L}}\text{C}_{\text{e}}}{\text{1+}\text{K}_{\text{L}}\text{C}_{\text{e}}}$ | (4) |
| --- | --- |
| $\text{R}_{\text{L}}\text{=}\frac{\text{1}}{\text{1+}\text{K}_{\text{L}}\text{C}_{\text{0}}}$ | (5) |
| $\text{Q}_{\text{e}}\text{=}\text{K}_{\text{F}}\text{C}_{\text{e}}^{\frac{\text{1}}{\text{n}}}$ | (6) |

where $\text{Q}_{\text{e}}$ is the adsorption capacity of adsorbent at equilibrium, (mg g^-1^); $\text{Q}_{\text{max}}$ is the maximum adsorption capacity, (mg g^-1^); $\text{C}_{\text{e}}$ is the concentration of dye at equilibrium after adsorption, (mg L^-1^); $\text{K}_{\text{L}}$ is the Langmuir constant (L mg^-1^); $\text{R}_{\text{L}}$ is the separation factor fitted by Langmuir isotherm model; $\text{C}_{\text{0}}$ is the initial dye concentration (mg L^-1^); $\text{K}_{\text{F}}$ is the Freundlich adsorption constant, (mg g^-1^); 1/n is the Freundlich heterogeneity factor.


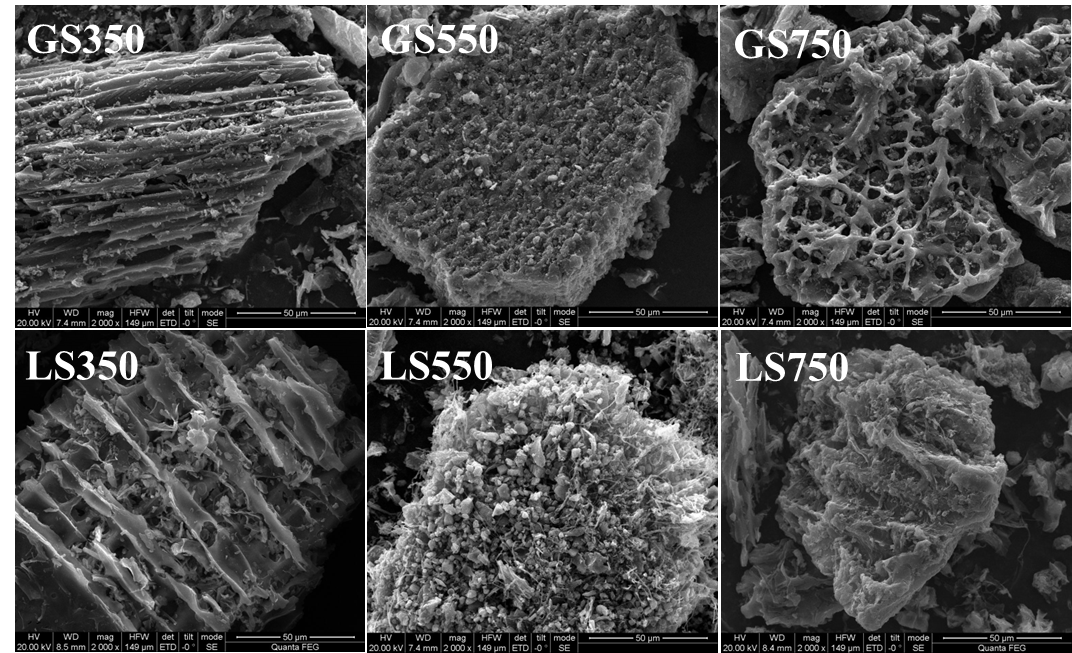


**Fig. S1.** SEM images of GSBC and LSBC (×2000 times).


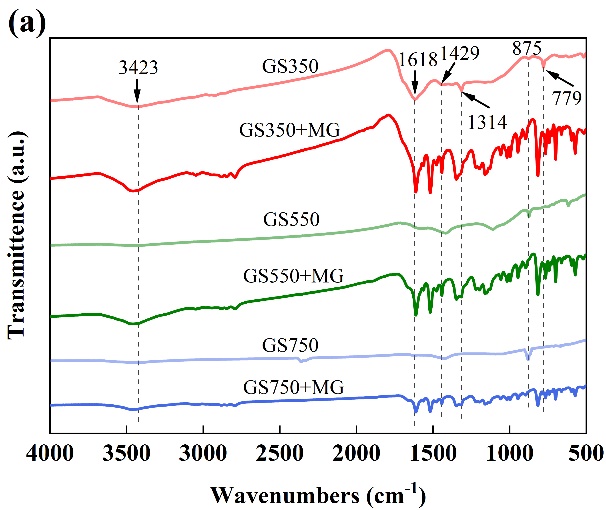

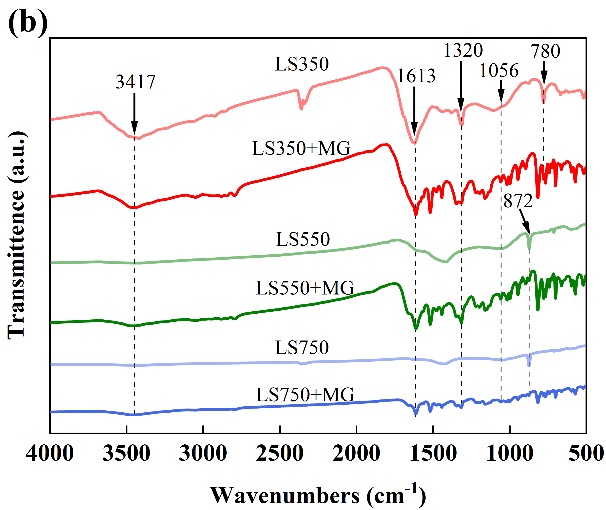

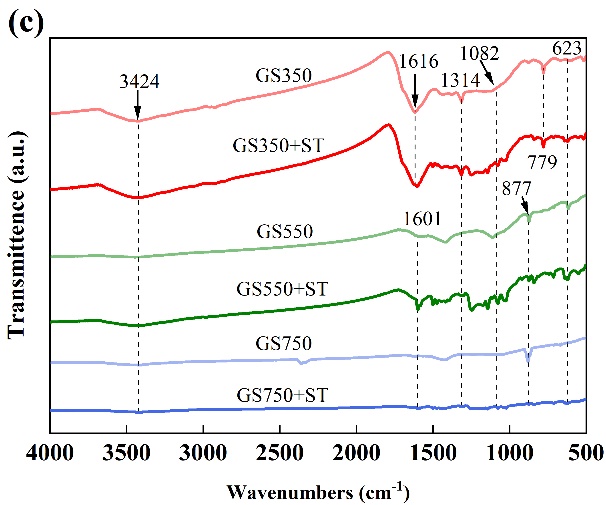

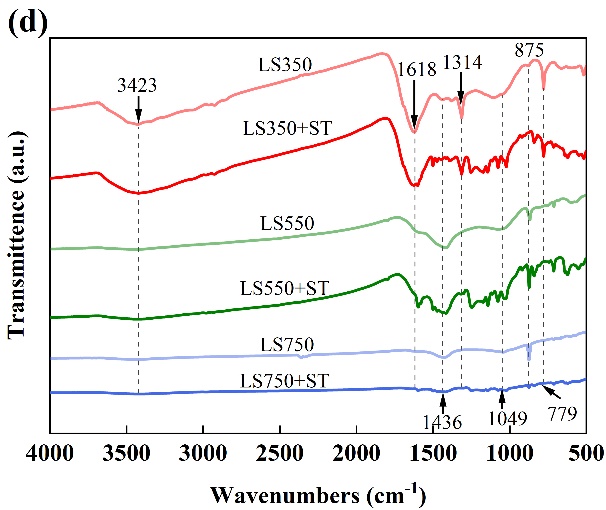


**Fig. S2.** FTIR spectra of GSBC and LSBC before and after adsorption of MG (a, b) and ST (c, d).

**Table S1.** Parameters of pseudo-first order and pseudo-second order kinetic models of MG and ST.

| Dyes | Samples | *Q*_e_^a^ (mg/g) | Pseudo-first order | | |  | Pseudo-second order | | |
| --- | --- | --- | --- | --- | --- | --- | --- | --- | --- |
|  |  |  | *Q*_e_^b^ (mg g^-1^) | *k*_1_ (min^-1^) | *R*^2^ |  | *Q*_e_^b^ (mg g^-1^) | *k*_2_ (min^-1^) | *R*^2^ |
| MG | GS350 | 6423.03±169.30 | 6522.14±178.67 | 0.0055±0.0005 | 0.991 |  | 7792.29±512.83 | 7.3059*10^-7^±1.9592*10^-7^ | 0.972 |
|  | GS550 | 7286.17±196.32 | 7315.11±178.69 | 0.0065±0.0005 | 0.991 |  | 8578.39±481.49 | 8.2029*10^-7^±1.9481*10^-7^ | 0.974 |
|  | GS750 | 8292. 37±182.54 | 8363.30±157.29 | 0.0083±0.0005 | 0.994 |  | 9575.06±472.65 | 9.9136*10^-7^±2.1725*10^-7^ | 0.973 |
|  | LS350 | 7452.72±128.31 | 7501.17±150.14 | 0.0113±0.0008 | 0.991 |  | 8420.52±424.10 | 1.5936*10^-6^±3.7435*10^-7^ | 0.964 |
|  | LS550 | 9330.77±124.76 | 9370.35±131.68 | 0.0194±0.0000 | 0.993 |  | 10170.35±391.84 | 2.5272*10^-6^±4.9577*10^-7^ | 0.965 |
|  | LS750 | 9332.45±146.98 | 9382.36±156.15 | 0.0124±0.0007 | 0.993 |  | 10458.72±485.92 | 1.4417*10^-6^±3.1734*10^-7^ | 0.959 |
| ST | GS350 | 1581.59±26.98 | 1494.87±34.26 | 0.0662±0.0073 | 0.898 |  | 1569.03±7.7359 | 7.0080*10^-5^±2.4958*10^-6^ | 0.996 |
|  | GS550 | 1670.48±19.36 | 1602.20±28.31 | 0.0832±0.0079 | 0.907 |  | 1669.41±8.3326 | 9.0574*10^-5^±3.73199*10^-6^ | 0.994 |
|  | GS750 | 1799.58±13.75 | 1734.77±34.45 | 0.1212±0.0161 | 0.728 |  | 1793.39±12.9618 | 1.4259*10^-4^±1.1216*10^-5^ | 0.972 |
|  | LS350 | 1987.77±39.65 | 1910.80±40.20 | 0.0478±0.0044 | 0.948 |  | 2018.73±19.00 | 3.6600*10^-5^±2.1785*10^-6^ | 0.992 |
|  | LS550 | 2448.68±19.05 | 2371.71±45.68 | 0.0892±0.0095 | 0.871 |  | 2467.73±9.93 | 6.7320*10^-5^±2.3368*10^-6^ | 0.996 |
|  | LS750 | 2573.31±11.00 | 2485.56±59.62 | 0.0941±0.0129 | 0.772 |  | 2584.45±20.15 | 6.9209*10^-5^±4.8291*10^-6^ | 0.981 |

Note: MG: MalachiteGreen oxalate; ST: Safranine T; a: actual adsorption capacity; b: Theoretical adsorption capacity; *k*_1_ and *k*_2_: kinetic constants of pseudo-first order and pseudo-second order; R^2^: correlation coefficient.

**Table S2****.** Parameters of intra-particle diffusion models of MG and ST.

| Dyes | Samples | Intra-particle diffusion | | | | | | | | |
| --- | --- | --- | --- | --- | --- | --- | --- | --- | --- | --- |
|  |  | k_p1_  (mg g^-1^ min^-1/2^) | k_p2_  (mg g^-1^ min^-1/2^) | k_p3_  (mg g^-1^ min^-1/2^) | c_1_ | c_2_ | c_3_ | R_1_^2^ | R_2_^2^ | R_3_^2^ |
| MG | GS350 | 413.79±26.80 | 20.35±12.75 | - | -1418.50±239.72 | 5754.06±343.82 | - | 0.979 | 0.718 | - |
|  | GS550 | 472.36±38.62 | 15.84±4.19 | - | -1355.32±345.46 | 6750.48±112.99 | - | 0.976 | 0.935 | - |
|  | GS750 | 563.14±46.08 | 2.33±2.06 | - | -1212.79±412.17 | 8217.20±55.60 | - | 0.967 | 0.562 | - |
|  | LS350 | 562.79±58.38 | 1.26±1.04 | - | -1061.07±522.19 | 7407.83±30.90 | - | 0.948 | 0.594 | - |
|  | LS550 | 695.76±77.82 | 0.84±0.67 | - | -1023.01±696.06 | 9300.68±19.97 | - | 0.940 | 0.611 | - |
|  | LS750 | 634.06±102.23 | 0.74±0.65 | - | 529.59±914.41 | 9305.85±19.25 | - | 0.882 | 0.572 | - |
| ST | GS350 | 171.31±28.68 | 21.56±5.76 | 2.64±0.02 | 292.47±128.25 | 1184.18±68.20 | 1481.21±0.60 | 0.973 | 0.933 | 0.999 |
|  | GS550 | 171.24±39.77 | 16.42±0.27 | 1.70±0.53 | 464.21±177.84 | 1379.62±3.15 | 1604.34±15.86 | 0.949 | 0.999 | 0.910 |
|  | GS750 | 115.47±20.39 | 12.45±3.92 | 0.82±0.12 | 950.63±91.18 | 1582.15±46.33 | 1768.15±3.62 | 0.970 | 0.910 | 0.978 |
|  | LS350 | 242.74±27.28 | 28.39±1.50 | 1.07±0.19 | 85.01±121.86 | 1475.50±17.72 | 1946.73±5.50 | 0.988 | 0.997 | 0.971 |
|  | LS550 | 232.29±21.08 | 28.86±3.03 | 0.12±0.11 | 821.75±94.27 | 1997.81±35.90 | 2444.38±3.32 | 0.992 | 0.989 | 0.543 |
|  | LS750 | 173.50±2.96 | 27.94±3.12 | 0.12±0.11 | 1182.93±13.22 | 2139.19±36.90 | 2569.01±3.32 | 0.999 | 0.988 | 0.543 |

Note: *k*_pi_ _(i=1, 2…)_: Intraparticle diffusion rate constant; c: the boundary layer constant; R^2^: correlation coefficient.

**Table S3.** Parameters of adsorption isotherm models of MG and ST.

| Dyes | Samples | Langmuir | | | |  | Freundlich | | |
| --- | --- | --- | --- | --- | --- | --- | --- | --- | --- |
|  |  | *Q*_max_ (mg g^-1^) | *K*_L_ (L mg^-1^) | *R*_L_ | *R*^2^ |  | *K*_F_ (mg g^-1^) | 1/*n* | *R*^2^ |
| MG | GS350 | 6488.00±880.59 | 0.0134±0.0076 | 0.0209±0.0037 | 0.883 |  | 827.61±121.74 | 0.3030±0.0202 | 0.986 |
|  | GS550 | 6815.78±959.87 | 0.0833±0.0675 | 0.0020±0.0010 | 0.819 |  | 1770.59±192.21 | 0.2317±0.0352 | 0.982 |
|  | GS750 | 7730.27±877.03 | 0.1542±0.0810 | 0.0018±0.0003 | 0.897 |  | 2001.82±167.74 | 0.2800±0.0225 | 0.988 |
|  | LS350 | 6854.55±700.89 | 0.0552±0.0285 | 0.0051±0.0013 | 0.892 |  | 1533.56±125.98 | 0.2399±0.0245 | 0.992 |
|  | LS550 | 9240.26±901.42 | 0.4375±0.0689 | 0.0007±0.0000 | 0.991 |  | 3687.08±189.72 | 0.5558±00117 | 0.989 |
|  | LS750 | 9388.07±1219.45 | 0.6552±0.1236 | 0.0004±0.0000 | 0.984 |  | 4779.46±376.17 | 0.5673±0.0225 | 0.950 |
| ST | GS350 | 3522.933±160.57 | 0.0003±0.0000 | 0.4878±0.0257 | 0.909 |  | 4.9456±0.6948 | 0.7244±0.0359 | 0.999 |
|  | GS550 | 3527.40±130.20 | 0.0003±0.0001 | 0.4878±0.0152 | 0.899 |  | 5.4567±0.8826 | 0.7166±0.0422 | 0.998 |
|  | GS750 | 4062.64±165.20 | 0.0003±0.0000 | 0.4878±0.0323 | 0.889 |  | 5.3090±0.7867 | 0.1884±0.0371 | 0.998 |
|  | LS350 | 2848.10±333.60 | 0.0022±0.0005 | 0.1149±0.0264 | 0.901 |  | 39.90±4.40 | 0.5743±0.0539 | 0.998 |
|  | LS550 | 3610.60±338.20 | 0.0028±0.0005 | 0.0926±0.0100 | 0.911 |  | 48.34±3.96 | 0.6062±0.0381 | 0.999 |
|  | LS750 | 3871.48±372.60 | 0.0035±0.0007 | 0.0755±0.0126 | 0.928 |  | 65.91±5.58 | 0.5875±0.0427 | 0.999 |

Note: *Q*_max_: maximum adsorbed amount; *K*_L_: Langmuir constant; *R*_L_: Separation factor; *K*_F_: Freundlich constant; 1/*n*: Freundlich heterogeneity factor; *R*^2^: correlation coefficient.
